# Glomangioma of the trachea: A case report and literature review

**DOI:** 10.3892/ol.2015.2871

**Published:** 2015-01-13

**Authors:** YANG TAN, PENG YANG, XIAOYU DENG, YAN TANG

**Affiliations:** 1 Department of Pathology, Dujiangyan Maternal and Child Health Care Hospital, Chengdu, Sichuan 611830, P.R. China; 2 Department of Pathology, The Third People’s Hospital of Chengdu, Chengdu, Sichuan 610031, P.R. China

**Keywords:** glomus tumor, glomangioma, trachea, segmental resection

## Abstract

A 44-year-old male presented with progressing cough, dyspnea and hemoptysis due to a tracheal tumor involving the posterior wall of the lower trachea, with severe airway obstruction and coagulopathy. Consequently the patient underwent segmental resection of the trachea with an end-to-end anastomosis. Twenty months after treatment there remained no evidence of endobronchial recurrence at bronchoscopy or imaging studies. The diagnosis was benign tracheal glomus tumor (GT) which is an exceedingly rare mass lesion in the trachea. There are three subtypes: GT proper, glomangioma and glomangiomyoma. The present study describes the clinical and pathological features of glomangioma through a case report and literature review. To the best of our knowledge, this is the fifth report of glomangioma subtype arising from the trachea.

## Introduction

Glomus tumors (GTs) are neoplasms arising from the modified smooth muscle cells surrounding arteriovenous anastomosis (1,2). GT is an uncommon soft tissue tumor with an incidence of 1.6%, which is usually located in the dermis and subcutaneous tissue, with ≤65% occurring in the subungual area. Due to sparse or absent glomus bodies in the visceral organs, extracutaneous presentation of GT is rarely observed (3–8). Previously reported atypical sites of origin include the stomach, mediastinum, vagina, penis, lung, patella and trachea. Histologically, GTs have been divided into three subtypes: Classic glomus tumors, glomangiomas, and glomangiomyomas. Glomangiomas are an uncommon type, accounting for <20% of GTs (1,2,9–11). Until now, only 27 cases of GTs, and five reports of glomangioma subtype arising from the trachea, including the present case, have been reported (8–32). GTs are usually benign and recurrence rates are variable, ranging from 10 to 30% (1,2). The present study reported a primary GT of the trachea, which is a possible differential diagnostic alternative when a tracheal tumor is detected by radiographic or endoscopic examination. Written informed consent was obtained from the patient and the patient’s family.

## Case report

**Figure 1:**
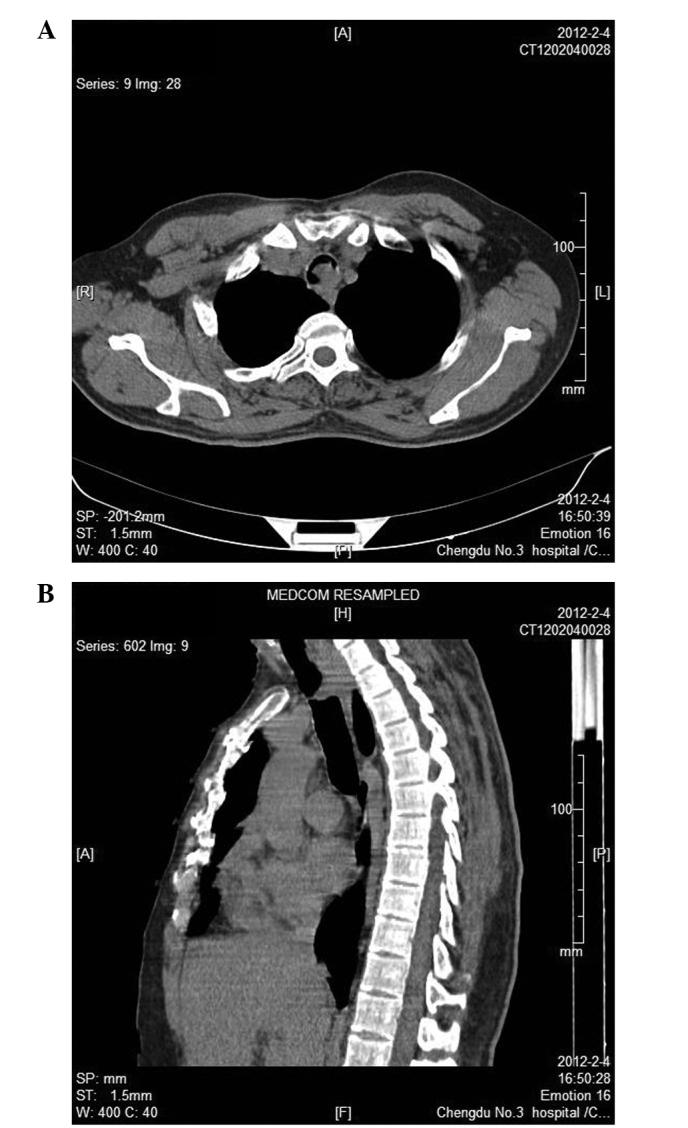
Computed tomograms of homogenous mass intratrachea at the layer of the superior border of the manubrium: (A) Transverse section and (B) median sagittal section of the chest.

A 44-year-old male, exhibiting a defined tracheal tumor that was diagnosed by the local hospital (Bazhou People’s Hospital, Bazhou, China) two months earlier, was admitted to our hospital (The Third People’s Hospital of Chengdu, Chengdu, China) due to acute respiratory distress. The patient had suffered from cough, expectoration and dyspnea without any evident incentive for >1 year. Six days before admission, the symptoms were aggravated with hemoptysis. A chest X-ray scan was found to be normal. However, a computed tomographic (CT) scan revealed a demarcated homogenous intratracheal mass at the layer of the superior border of the manubrium (Fig. 1). Flexible bronchoscopy revealed a sessile tumor with a smooth surface arising from the posterior wall of the trachea, which occluded ~90% of the trachea lumen, at 3 cm proximal to the carina. A biopsy was performed to elucidate the nature of the tumor. At 3 h after referral, the patient exhibited no response to voice and became signally dyspneic and cyanotic in the face and extremities, which indicated apnea induced by neoplastic obstruction.

To prevent mortality and restore the airway, endotracheal intubation was conducted initially using a rigid tracheoscope under surface anesthesia. Due to the unstable condition of the patient, comprehensive treatment was administered to prepare for the operation. A preoperative coagulation profile revealed prolonged activated partial thromboplastin time (APTT; 68.3 sec) and prothrombin time (PT; 19.1 sec), which indicated an increased risk for the surgery. With plasma transfusion, after five days the patient underwent segmental resection of the trachea with an end-to-end anastomosis and the bilateral pulmonary infection led to a poor general postoperative condition. Due to being bed-ridden postoperatively, 13 days following the surgery the patient exhibited femoral and deep vein thrombosis. Thrombolytic therapy was challenging, due to the persistent bleeding disorder. To prevent pulmonary embolus, vena cava filters were placed and clot-busting therapy was conducted simultaneously. The patient was discharged without the support of ventilation 26 days after presentation. Three months after resection, the patient underwent follow-up with CT and endoscopy to exclude focal recurrence or suspicious metastasis. The lumen of the trachea remained clear for 20 months after the surgery.

**Figure 2:**
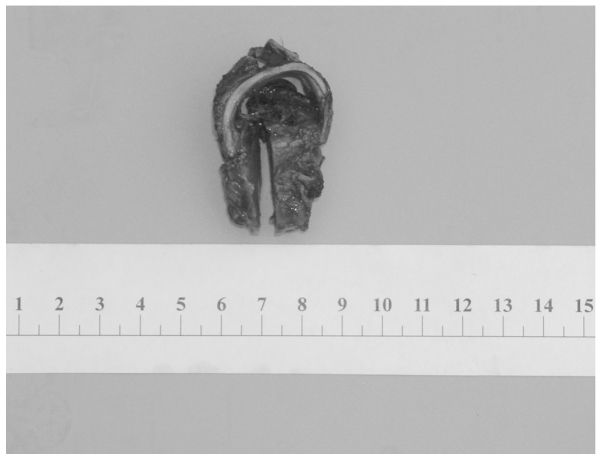
Resected tracheal specimen exhibits the mass arising from the posterior wall of the trachea.

**Figure 3:**
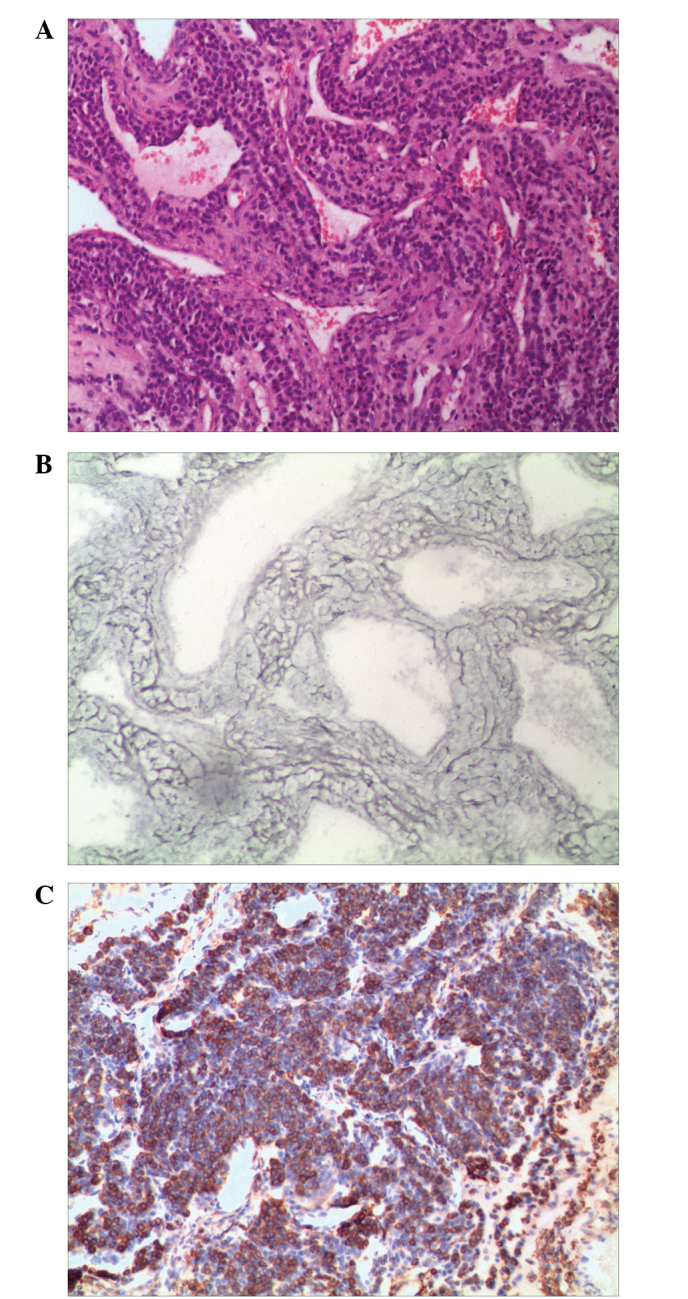
(A) Uniform polygonal cells with round to ovoid nuclei and slightly eosinophilic cytoplasm surrounded by dilated capillary-sized vessels (hematoxylin and eosin; magnification, ×200). (B) Reticulin stain revealed a delicate network of reticulum fibers lying between individual tumor cells (magnification, ×200). (C) On immunohistochemical evaluation, tumor cells were stained for smooth muscle actin (magnification, ×200).

The surgical specimen was ~2 cm in length, with a luminal diameter of 2.5 cm. The specimen exhibited a 3×2.5×1 cm red-brown mass sessile in the posterior wall of the trachea, which occupied the lumen as a polypoid mass (Fig. 2). Microscopically, the tumor was composed of epithelioid round to polygonal cells with defined cellular borders, weakly eosinophilic or clear cytoplasm and uniform round to ovoid nuclei. These formed solid sheets, small nests or organoidal structures surrounding dilated and tangled venous vessels, which were different from the thin-walled and capillary-like vascular channels normally observed in GT proper type. Cellular atypia and mitotic figures were absent (Fig. 3A). Gomori’s staining demonstrated a delicate network of reticulum fibers lying between individual tumor cells (Fig. 3B). In addition, evidence of squamous metaplasia was observed in the intact overlying respiratory epithelium. Immunohistochemical staining revealed positivity for vimentin and smooth muscle actin antibodies (Fig. 3C). Pan-cytokeratin, desmin, chromogranin, synaptophysin, S-100, calfetinin, leukocyte common antigen, HMB-45 and cluster of differentiation 99 were negative (Table I). The tumor was diagnosed as a glomangioma.

## Discussion

GTs are uncommon mesenchymal neoplasms. The etiology of these tumors remains a conundrum, and certain individuals attribute GTs to trauma, endocrine disorder or autosomal dominant inheritance (1,2). Murray and Stout (2) indicated that GT often occurred in subungual hematoma and the fingertips of female patients, while in the extradigital tissues of male patients. This viewpoint coincides with previous studies on the tracheal GTs presented in Table II, in which the majority of cases involved male patients. GTs in the trachea are scarce. The first GT case was reported in 1950 and, to the best of our knowledge, since then only 27 additional cases (including the present study) have been reported (Table II) (8–32). Based on the literature reviewed (Table III), the majority of cases have been identified on the posterior wall of the lower two-thirds of the trachea (82%), where mucus glands and vessels are numerous. The cases included 16 males and 10 females (1.6:1), with a mean age of 50. The main symptoms included coughing, dyspnea and hemoptysis. Certain patients also suffered from chest pain, stridor and hoarseness. The mass diameter had a range of 1.2–4.5 cm. Almost all the tumors, including that of the present study, were benign and noninvasive.

To the best of our knowledge, no malignant GTs have been described in the trachea thus far. The histological morphology varies in subtypes according to the relative proportions of the glomus cells, vascular structures and smooth muscle tissue (2,33). In addition to the classic three subtypes, including GT proper, glomangioma and glomangiomyoma, an oncocytic variant exists that was first described in 1990 (13). GT proper type accounts for ~75% of all the GTs, which also applies to the tracheal mass, while glomangioma occupies 20% of all the cases (Table II). The oncocytic tumors are the least common, comprising 1/20 of all the tracheal GTs reported in the literature (13). In the present study, the tumor was characterized by organoid nests and solid sheets of uniform round or polygonal cells around the venous vessels, but not thin-walled or capillary-like vascular channels. This is the fifth description of a glomangioma arising from the trachea. In the study by Shin *et al*, the round glomus cells represented the largest component of tumor cells surrounding the thin-walled vascular spaces, leading to the hypothesis that this may be a subtype of glomangioma (13). Based on the Pathology and Genetics of Tumors of Soft Tissue and Bone (World Health Organization Classification of Tumors), the main histological characteristic of glomangioma is that the glomus cell clusters are arranged around dilated venous vessels (33). The immunohistochemical features have contributed to differential diagnosis with carcinoid and hemangiopericytoma (28,32,33). GTs are positive for vimentin and smooth musle actin, but negative for neuroendocrine and epithelial markers, including S-100 protein, chromogranin, desmin, cytokeratins and factor VIII.

To avoid local recurrence, tracheal resection is widely used and exhibits favorable prognosis (Table II). Endoscopic intervention alone is also applied in a limited number of cases, which has rigorous indications if: The lesion is strictly confined without extension; histology confirms the tumor is benign; as a temporary measure for surgery preparation; or the patient is not fit or willing to undergo surgical resection. In addition, interventional bronchoscopy is a first-line treatment to immediately restore the airway patency in urgent situations (14,16,17, 25–27,30).

Furthermore, as an uncommon site, treating the complications and accompanying diseases during the perioperative period is crucial in order to increase survival rates. The main cause of mortality reported in the literature following surgery was not the primary tumor or the procedure but the complications (9,10). The present patient exhibited a severe clinical condition and the symptoms were caused by tumor obstruction. Therefore, endotracheal intubation was performed to aid the recovery of the stabilization, ensuring that the patient fit for the procedure. Due to his severe coagulopathy, preoperative correction of coagulation disturbance was performed to prepare for the surgery. The filters of the vena cava and warfarin administration were arranged simultaneously following surgery to treat femoral and deep vein thrombosis caused by a long time period in bed. Active sputum excretion, prevention of infections and early mobilization is beneficial for recovery.

In conclusion, previous studies and the present report reveal that the incidence of GTs in the trachea is low and these tumors primarily occur in males, with unknown etiology and concealed onset. Patients with GTs usually resort to medical attention for symptoms of airway obstruction. CT imaging, magnetic resonance imaging and flexible bronchoscopy of GTs reveal a lumen-occupying lesion. Surgical management is generally the primary option and histopathological examination may determine the diagnosis. The long-term prognosis of the disease is promising.

## Figures and Tables

**Table I:** Antibody panel used in this case study.

Antibody/molecule	Clone	Dilution	Expression
SMA	1A4	1:70	(+)
Vimentin	SP20	1:70	(+)
Desmin	D33	1:70	(−)
Pan-cytokeratin	AE1/AE3	1:70	(−)
Chromogranin	SP12	1:70	(−)
Synaptophysin	SP11	1:70	(−)
S-100	4C4.9	1:70	(−)
Calfetinin	Polyclone	1:60	(−)
LCA	ZB11+PD7/26	1:70	(−)
Melanosome	HMB-45	1:70	(−)
CD99	SP119	1:70	(−)

Antigen retrieval was PH9.0EDTA (microwave), and Zeta Corporation (Sierra Madre, CA, USA) provided the mouse anti-human monoclonal antibodies for the present study. (+), positive; (−), negative; CD99, cluster of differentiation 99; SMA, smooth muscle actin; LCA, leukocyte common antigen.

**Table II:** Reported cases of tracheal glomus tumor.

First author, year (ref)	Patient age/gender	Symptoms	Size (cm)	Tracheal location	Main treatment	Follow-up
Hussarek, 1950 (8)	43/F	Dyspnea	‘Bean-sized’	Upper trachea	Tracheal resection	Not stated
Fabich, 1980 (9)	63/M	Cough	2.5×2×1	Lower trachea	Tracheal resection	Succumbed to complications, postop day 10
Heard, 1982 (10)	50/M	Asthma-like symptoms	2.5×1.5×1.0	Lower trachea	Tracheal resection	Sepsis, died postop day 15
Ito, 1988 (11)	51/M	Respiratory infections, hemoptysis	1.5×1.2×1.0	Upper trachea	Tracheal resection	No recurrence at 2 years
Kim, 1989 (12)	54/F	Cough, dyspnea, hemoptysis	1.5×1.2	Mid-trachea	Tracheal resection	No recurrence at 13 months
Shin, 1990 (13)	47/F	Intermittent cough and hemoptysis	1.5×1.0×1.0	Lower trachea	Tracheal resection	Not stated
Garcia-Prats, 1991 (14)	58/M	Dyspnea, cough, hemoptysis	2.5×1.8	Mid-trachea	Tracheal resection	No recurrence at 8 months
Haraguchi, 1991 (15)	61/M	Asymptomatic	1.2	Mid-trachea	Tracheal resection	Not stated
Arapantoni, 1995 (16)	65/M	Dyspnea, hemoptysis	4.5×3	Lower trachea	Endoscopic resection and Nd-YAG	No recurrence at 1 year
Koskinen, 1998 (17)	66/M	Asymptomatic	3×2	Lower trachea	Endoscopic resection, Nd-YAG, and external radiotherapy	Not stated
Watanabe, 1998 (18)	43/M	Hoarseness	2.0×1.6×1.4	Lower trachea	Tracheal resection	No recurrence at 20 months
Menaissy, 2000 (19)	34/M	Hemoptysis	2.4×2.1×1.6	Mid-trachea	Tracheal resection	No recurrence at 4 months
Gowan, 2001 (20)	73/M	Cough, hemoptysis, dyspnea, Chest pain	1.6×0.6×0.3	Mid-trachea	Tracheal resection	No recurrence at 6 years
Chien, 2003 (21)	50/F	Cough, dyspnea, hemoptysis	2.5×2.5×2	Lower trachea	Tracheal resection	No recurrence at 1 years
Nadrous, 2004 (22)	39/M	Intermittent hemoptysis	2.0×1.5×1.5	Upper trachea	Tracheal resection	No recurrence at 3 months
Haver, 2008 (23)	10/F	Dyspnea	1.8×1.3×1.3	Mid-trachea	Tracheal resection	No recurrence at 2 years
Colaut, 2008 (24)	70/M	Dyspnea	2×1×1	Mid-trachea	Endoscopic resection and Nd-YAG	No recurrence at 2 years
Parker, 2010 (25)	43/F	Chest pain, asthma	2.0×1.6×1.5	Lower trachea	Tracheal resection	Not stated
Shang, 2010 (26)	59/M	Chest pain, dyspnea	2.0×1×0.5	Lower trachea	Endoscopic resection	No recurrence at 12 months
	22/F	Cough, dyspnea	1.8×1.5×1.4	Lower trachea	Endoscopic resection	No recurrence at 12 months
Sakr, 2011 (27)	66/M	Dyspnea, stridor	2×1.2×0.8	Upper trachea	Endoscopic resection and tracheal resection	No recurrence at 21 months
Mogi, 2011 (28)	56/F	Cough, dyspnea	1.3×1.2×1.1	Lower trachea	Tracheal sleeve resection	No recurrence at 9 months
Okereke, 2011 (29)	58/M	Stridor, shortness of breath.	1.1	Mid-trachea	Tracheal resection	Not stated
Norder, 2012 (30)	49/F	Cough, dyspnea	1.2×1.1×1.1	Upper trachea	Endoscopic resection	Not stated
Lang-Lazdunski L, 2012 (31)	62/F	Cough, dyspnea	1.6	Lower trachea	Left upper sleeve lobectomy	Not stated
Fan, 2013 (32)	15/M	Cough, hemoptysis	2.5	Lower trachea	Tracheal resection	No recurrence at 12 months
Present study	44/M	Cough, hemoptysis, dyspnea	3×2.5×1	Lower trachea	Tracheal resection	No recurrence at 20 months

F, female; M, male; Nd-YAG, neodymium-yttrium-aluminum-garnet laser; postop, postoperative.

**Table III:** Summary characteristics of previously reported patients with glomus tumor of the trachea^a^.

Characteristic	Value
Age, range (mean)	10–73 (50)
Male:Female	1.6:1
Symptoms, n (%)^b^	
Cough	11 (41)
Hemoptysis	11 (41)
Dyspnea	15 (56)
Chest pain	3 (11)
Asthma or stridor	3 (11)
None	2 (7)
Tumor size, range (mean)	1.1–4.5 (2.06)
Tumor location, n (%)^b^	
Upper trachea	5 (18%)
Mid-trachea	8 (30%)
Lower trachea	14 (52%)
Treatment, n	
Tracheal resection	19
Endoscopic resection	7
Other	1

a Data were obtained from references 8–32.

b Certain patients presented multiple symptoms: Symptoms (%), number of patients with this symptom/total number of patients × 100; tumor location (%), number of patients in this location/total number of patients × 100.
